# Acid-mediated extraction of gelatine from chicken by-products: impact on yield, molecular characteristics, and structural-thermal properties

**DOI:** 10.3389/fchem.2026.1787144

**Published:** 2026-06-23

**Authors:** Omaima Aidat, Mohamad Khairi Zainol, Ilham Ben Amor, Zamzahaila Mohd Zin, Humam Shaaban Barhoum, Huda Alsaeedi, Mikhael Bechelany, Ahmed Barhoum

**Affiliations:** 1 Department of First Cycle in Natural and Life Sciences, Higher National School of Forests (ENSF), Khenchela, Algeria; 2 Faculty of Fisheries and Food Science, University Malaysia Terengganu, Kuala Terengganu, Malaysia; 3 Department of Process Engineering and Petrochemical, Faculty of Technology, University of El Oued, El Oued, Algeria; 4 Laboratory of Valorisation and Technology of Saharian Resources (VTRS), Department of Process Engineering and Petrochemistry, Faculty of Technology, University of El Oued, El Oued, Algeria; 5 Department of Plant Protection, Faculty of Agriculture, Damascus University, Damascus, Syria; 6 Department of Chemistry, College of Science, King Saud University, Riyadh, Saudi Arabia; 7 Institut Européen des Membranes, IEM, UMR-5635, University Montpellier, ENSCM, CNRS, Montpellier, France; 8 Functional Materials Group, Gulf University for Science and Technology (GUST), Hawally, Kuwait; 9 School of Chemical and BioPharmaceutical Sciences, Technological University Dublin, Dublin, Ireland; 10 Nanolab Research Centre, Physical to Life Sciences Research Hub, Technological University Dublin, Dublin, Ireland

**Keywords:** acetic acid concentration, chicken by-products, gelatine extraction, molecular weight distribution, structural characterization, sustainable biopolymers, thermal properties

## Abstract

Despite the growing interest in utilizing poultry processing by-products as sustainable sources of gelatine, the influence of extraction conditions on gelatine quality, structure, and functionality remains insufficiently understood. This study investigated the effect of acetic acid concentration on the yield, molecular characteristics, and structural-thermal properties of gelatin extracted from chicken feet and heads. Gelatine was extracted using 3 v/v% (CBBG3) and 5 v/v% (CBBG5) acetic acid. CBBG5 produced a higher extraction yield (13.2%) than CBBG3 (10.49%), indicating enhanced collagen solubilization at higher acid concentration. However, CBBG5 also showed higher ash content (4.45 w/w% vs. 2.40 w/w%) and more pronounced yellow-green coloration. Structural characterization using FTIR, SEM, SDS-PAGE, and DSC revealed notable differences between the samples. FTIR spectra showed characteristic gelatine amide bands, with CBBG3 exhibiting stronger amide I and II signals, suggesting better preservation of molecular structure. SEM analysis revealed a smoother, more compact morphology for CBBG3, whereas CBBG5 displayed a rougher and more irregular surface. SDS-PAGE showed higher molecular weights of the α1 and α2 chains in CBBG3 (156 and 130 kDa, respectively) than in CBBG5 (137.1 and 82.6 kDa), indicating lower molecular degradation. DSC further confirmed the superior thermal stability of CBBG3, which exhibited a higher glass transition temperature (26.97 °C) and transition enthalpy (2.688 J g^-1^). Although increasing acetic acid concentration improved gelatin yield, extraction with 3 v/v% acetic acid resulted in superior molecular organization, structural integrity, and thermal stability. These findings highlight the importance of optimizing extraction conditions to balance yield and quality and demonstrate the potential of chicken by-products as a sustainable source of high-quality gelatine for food, pharmaceutical, and biomedical applications.

## Graphical Abstract

**Figure:**
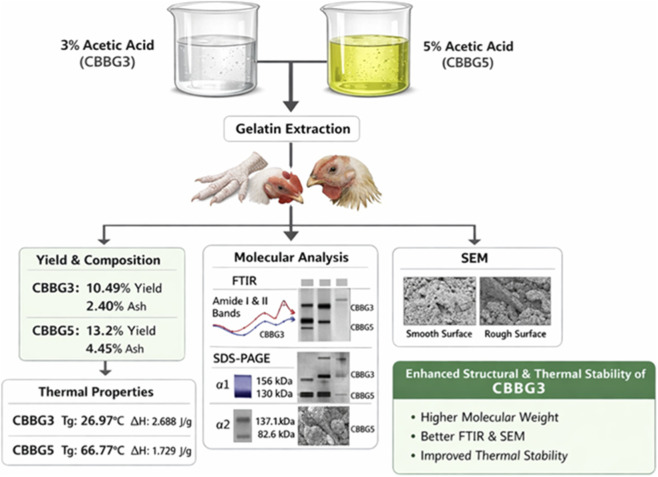

## 1 Introduction

Waste animal by-products, such as bones, hides, and connective tissues, are increasingly recognized as valuable sources of gelatine. Gelatine, a protein derived from collagen, is predominantly obtained from these by-products, making them an essential resource for various industries. The global gelatine market reflects this importance, with a projected growth rate of 6.5% CAGR from 2022 to 2030, driven by increasing applications in food, pharmaceuticals, and cosmetics (Alipal et al., 2021). Specifically, chicken by-products, including feet and heads, are rich in collagen and have been effectively used for gelatine extraction. For example, chicken feet alone are reported to contain approximately 30% collagen (Abedinia et al., 2020), which can be converted into gelatine through proper extraction processes. Using these by-products not only contributes to waste reduction but also supports sustainability in the food industry by repurposing materials that would otherwise be discarded. This approach aligns with the broader trend of using agricultural and food industry waste, transforming it into economically valuable products while minimizing environmental impact (Kim et al., 2020).

Gelatine is extracted from various animal sources, each providing unique properties suitable for different applications. For instance, pork and beef gelatines are widely used in the food industry due to their gelling and binding properties, essential for products like marshmallows and gelatine desserts (Rather et al., 2022a). Fish gelatines (derived from fish skins and bones) are prized for their lower allergenicity and are often preferred in dietary supplements and certain food products targeting specific consumer groups (Matulessy et al., 2021). In contrast, poultry-based gelatines, such as those from chicken, are increasingly being explored for their functional properties and cost-effectiveness. Chicken gelatines offer advantages in terms of lower fat content and potentially higher collagen yield from by-products such as feet and heads (Erg et al., 2018). Each type of gelatine has distinct thermal and gelling properties, making it suitable for specific applications. For example, beef gelatine has higher bloom strength, making it ideal for confectioneries, while fish gelatine’s solubility makes it suitable for dietary supplements and pharmaceuticals (Rather et al., 2022b). Understanding these differences allows for better selection of gelatine types based on desired properties for various industrial and consumer applications.

Extracting gelatine from chicken by-products involves several critical steps, each influencing the final gelatine’s quality. The process begins with pretreatment to eliminate non-collagenous materials and enhance collagen accessibility. Typically, alkaline solutions like 0.5 M NaOH are employed to break down connective tissues and prepare the material for subsequent acid extraction (Mokrejš et al., 2021). Alternatives such as Ca(OH)_2_ and KOH can also be used, with each offering varying levels of effectiveness depending on the waste material and desired gelatine characteristics (Alipal et al., 2021). Following this pretreatment, the material is soaked in an acid solution to dissolve the collagen. Acetic acid (1-5v/v%) is widely employed for collagen pretreatment and gelatine extraction (Abedinia et al., 2020). Alternative mineral acids have also been used. HCl is generally applied at concentrations of 0.1v/v% to 1v/v%, while H_2_SO_4_ is used at lower concentrations to avoid excessive collagen degradation (Pra et al., 2022). In contrast, organic acids such as citric acid and lactic acid offer milder extraction conditions and enable greater control over collagen hydrolysis (He et al., 2022). Following acid treatment, gelatine is solubilized through thermal extraction, typically by heating the system at temperatures between 75 °C and 80 °C to promote collagen denaturation and gelatine release (Matulessy et al., 2021). The gelatine solution is then dried and ground into powder. Research indicates that varying acid concentrations significantly affect gelatine’s molecular weight and functional properties (Erg et al., 2018). This method effectively uses waste by-products and produces high-quality gelatine suitable for various industrial applications.

Gelatin structure and functionality are strongly influenced by extraction conditions. Acid pretreatment is particularly important, as it governs collagen swelling, solubilization, and molecular degradation. Changes in acid concentration can therefore significantly affect yield, molecular weight distribution, and functional performance of the resulting gelatin. This study investigates the effect of acetic acid concentration on gelatin extracted from chicken by-products (feet and heads), aiming to optimize extraction efficiency while improving structural integrity, thermal stability, and functionality. Although poultry by-products are an abundant collagen source, systematic studies on how controlled acid concentrations influence the molecular, structural, and thermal properties of gelatin remain limited. In particular, the combined effect of alkaline pretreatment and acid extraction has not been fully explored. Acetic acid was selected for its efficiency in collagen conversion while minimizing excessive degradation. Different concentrations were applied to assess their impact. NaOH pretreatment was used to remove non-collagenous proteins and induce fiber swelling, enhancing acid penetration during extraction. Gelatin quality was evaluated using FTIR, SEM, SDS-PAGE, and DSC to assess functional groups, morphology, molecular profile, and thermal behavior. This study provides insight into structure–property relationships of poultry-derived gelatin, supporting process optimization and sustainable use of agro-industrial by-products for food, pharmaceutical, and biomaterial applications.

## 2 Experimental

### 2.1 Materials and reagents

Fresh chicken heads and feet (Arbor Acres broiler) were collected from a local slaughterhouse and meat processing facility, Poultry Group-West Algeria-Mostaganem (GAO-ORAVIO), located in the western region of Algeria. The samples were cleaned (de-nailed, plucked), and rinsed with tap water prior to transport to the laboratory. Equal amounts of chicken feet and head samples (50 g each) were chopped into small pieces (approximately 5 cm in size) and stored in plastic bags at −20 °C until gelatine extraction. The use of mixed chicken heads and feet was intended to better simulate real industrial by-product streams, where separation of anatomical parts is often not performed. This approach provides a more realistic assessment of process scalability and gelatine performance under practical production conditions. Food-grade acetic acid was obtained from Sigma-Aldrich (USA). All other chemicals and reagents used were of analytical grade and procured from certified suppliers.

### 2.2 Gelatine extraction from chicken by-product

**FIGURE 1:**
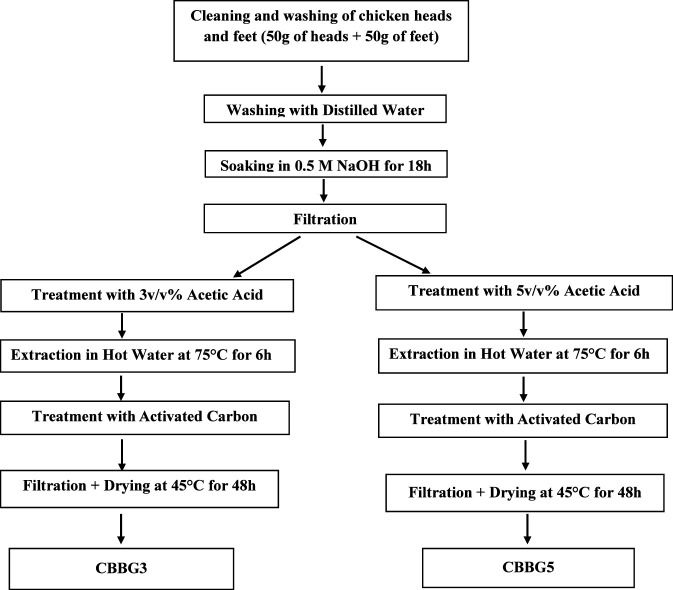
Illustration of gelatine extraction from a mixture of chicken feet and heads.

Gelatine was extracted from a mixture of chicken by-products, consisting of feet and heads (totalling 100 g per sample). The thawed chicken by-products were first subjected to deproteinization by soaking in a 0.5 M NaOH solution at a ratio of 1/10 (w/v) to remove non-collagenous materials (Aidat et al., 2023). The samples were then rinsed six times with 1 litter of distilled water per rinse, with each rinse involving 30 min of agitation to ensure thorough contact. This process was repeated until the pH reached 7, after which the samples were filtered. Following deproteinization, the samples were soaked in acetic acid at two different concentrations (3v/v% and 5v/v%) for 18 h at 4 °C, resulting in two distinct gelatine types: CBBG3 (treated with 3v/v% acetic acid) and CBBG5 (treated with 5v/v% acetic acid). For the acetic acid treatment, 30 mL or 50 mL of acetic acid was added and diluted with distilled water to a total volume of 1 L. The gelatine extraction was carried out by heating the samples in water in a water bath (Memmert WTB) at 75 °C for 6 h. The extract was then stirred with 4 g of activated carbon in 1 L of water for 20 min to remove odours and impurities. The final gelatine extract was filtered using vacuum filtration avec un papier filter N:4, poured into Petri dishes, and dried in a universal oven (Memmert) at 45 °C for 48 h. The dried gelatine sheets were ground into powder using an electric knife mill (Grindomix GM200 with a 1 L plastic container) and stored in plastic bags at room temperature for further analysis (see Figure 1). The yield of each extraction was calculated using the formula:

$$\text{Yield }\left(\%\right)=\left(\text{dry weight of gelatine}/\text{raw material weight}\right)\times 100.$$

### 2.3 Ash content and colour analysis of dried gelatine

The ash content was determined following the AOAC (2000) method. Briefly, the sample was incinerated in a muffle furnace at 550 °C, and the ash content was measured gravimetrically after the complete combustion of the sample. For colour measurement, 6.67% (w/v) gelatine gels were prepared. The colour parameters L*, a*, and b* were measured using a Chroma Meter (CR-400 model, Konica Minolta Sensing, Japan). These values allowed for the characterization of colour variations in the samples based on the acetic acid concentration used.

### 2.4 Fourier transform infrared spectroscopy (FTIR)

Gelatine powder was mixed with potassium bromide (KBr) in a 1/6 (w/w) ratio and ground using a mortar. The resulting mixture was compressed into a pellet using a hydraulic press and placed in the IR sample holder of the FTIR apparatus. The FTIR spectra were recorded in the wavenumber range of 500–4,000 cm^-1^ with a resolution of 4 cm^-1^, averaging 32 scans per sample (Zaater et al., 2024).

### 2.5 Scanning electron microscopy (SEM)

The microstructure of CBBG3 and CBBG5 gelatines was examined using SEM following the methods outlined by Wolf and Baker and Pieniazek and Messina (Pieniazek and Messina, 2016). The powdered or freeze-dried gelatine samples were mounted on the surface of an adhesive sticker attached to the specimen holder. Prior to SEM analysis (Jeol-6360, USA), the samples were sputter-coated with a thin layer of 99% pure gold using an Auto Fine Coater. SEM images were captured at a magnification of 5,000x to visualize the microstructural details of the gelatine samples.

### 2.6 SDS-PAGE gel electrophoresis

Sodium dodecyl sulphate-polyacrylamide gel electrophoresis (SDS-PAGE) was performed to analyse the molecular weight distribution of the gelatine samples, following the method of Junjie (2019), with minor modifications. Gelatine solutions (5 mg/mL protein concentration) were prepared and diluted 1:1 (v/v) with a sample buffer containing β-mercaptoethanol. The solutions were heated at 90 °C for 5 min. Aliquots of 10 µL of the prepared samples (2.5 mg/mL) and molecular weight markers were loaded onto ready-to-use 4%–20% gradient gels (Bio-Rad Laboratories, Hercules, CA). Electrophoresis was conducted at a constant voltage of 120 V using a standard electrophoresis unit. Following electrophoresis, the gels were stained with Coomassie Brilliant Blue R-250 and destained using a solution of acetic acid, methanol, and distilled water (50:40:10, v/v/v). The gels were scanned and imaged using the Amersham Biosciences Image Scanner (Uppsala, Sweden).

### 2.7 Differential scanning calorimetry (DSC)

DSC analysis was performed using a Perkin Elmer DSC 8000 device to evaluate the thermal properties of the gelatine samples. Four milligrams of freeze-dried gelatine powder were heated from −20 °C to 200 °C at a rate of 5 °C/min under a nitrogen gas atmosphere, as described in (Esmaeili‐Kaliji et al., 2025).

### 2.8 Statistical analysis

Statistical analyses were conducted using Minitab v.18. Data were compared statistically through one-way analysis of variance (ANOVA). Differences between the two gelatine samples were assessed using a T-test, with a significance level set at p ≤ 0.05.

## 3 Results and discussion

### 3.1 Yield, ash content and colour

The gelatine yield was significantly higher in the CBBG5 sample (13.2%) compared to CBBG3 (10.49%) (Table 1). Using a higher concentration of acetic acid (5v/v%) in the CBBG5 extraction process likely enhanced collagen breakdown, resulting in greater gelatine recovery. This increase in yield suggests that stronger acidic conditions are more effective in promoting gelatine extraction from chicken by-products.

**TABLE 1:** Characteristics of the prepared gelatines at different acetic acid concentrations.

Parameter		CBBG3	CBBG5	P-value
Yield		10.49 ± 0.49	13.2 ± 0.24	<0.000
Ash (%)		2.40 ± 0.57	4.45 ± 0.69	0.333
CIELAB colour space	L*	61.14 ± 0.31	61.59 ± 0.27	0.28
	a*	−1.93 ± 0.24	−2.40 ± 0.04	0.05
	b*	9.76 ± 0.23	11.74 ± 0.37	<0.000

Ash content, which reflects the amount of inorganic minerals in the gelatine, also varied between the two samples. CBBG5 exhibited a higher ash content (4.45%) than CBBG3 (2.40%), indicating that the stronger acid environment improved gelatine extraction and led to greater mineral extraction (Table 1). The higher ash content in CBBG5 could affect the overall properties of the gelatine, including its applications.

In terms of colour, the differences in ash content and acid concentration during extraction played a significant role. CBBG5, with its higher mineral content, showed a more pronounced yellow (b* value) and greenish (a* value) hue compared to CBBG3 (Table 1). The increased mineral concentration in CBBG5, combined with the stronger acid treatment, likely altered protein-mineral interactions, resulting in a distinct greenish-yellow appearance.

### 3.2 Chemical composition and functional groups

**FIGURE 2:**
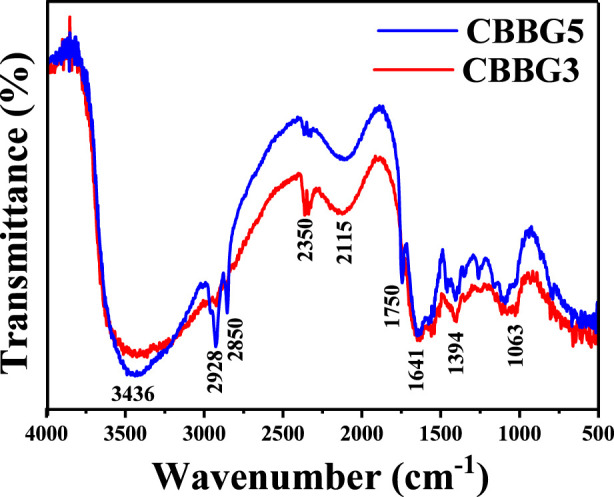
Representative FTIR spectra of gelatine extracted from a mixture of chicken by-products (feet-heads).

FTIR spectroscopy is a key technique for analysing gelatine’s chemical composition and structural stability. It detects vibrational changes in functional groups, especially the amide bands linked to protein secondary structure (Stani et al., 2020). In this study, FTIR was used to characterize two gelatine samples, CBBG3 and CBBG5. These were extracted using 3 v/v% and 5 v/v% acetic acid, respectively (see Figure 2). Both spectra showed strong absorption peaks in the characteristic amide regions. These peaks are essential for gelatine identification and structural evaluation.

The Amide I band is mainly associated with C=O stretching vibrations. It also includes minor contributions from N-H bending. This band appeared at 1,631 cm^-1^ for CBBG3 and 1,647 cm^-1^ for CBBG5. The shift indicates differences in secondary structure caused by acetic acid concentration. The Amide I band is sensitive to protein conformation. It reflects changes in molecular ordering and structural integrity (Ata et al., 2024; Cao et al., 2023).

The Amide II band is attributed to N-H bending and C-N stretching vibrations. It was observed at 1,559 cm^-1^ for CBBG3. In contrast, it shifted to 1,405 cm^-1^ for CBBG5. This strong shift suggests major changes in the gelatine backbone. It indicates a higher level of denaturation in CBBG5. It also suggests disruption of intermolecular interactions during extraction (Ata et al., 2024).

The Amide III band is associated with C-H bending and C-N stretching vibrations. It is linked to the triple-helix structure of collagen-derived gelatine. This band appeared at 1,243 cm^-1^ for CBBG3 and 1,259 cm^-1^ for CBBG5. The shift indicates partial loss of triple-helix structure in both samples. This is consistent with acid-induced conversion of collagen into amorphous gelatine (Chen et al., 2023).

In the higher wavenumber region (3,100–3,500 cm^-1^), a broad absorption band was observed in both samples. This band corresponds mainly to the Amide A band, arising from N-H stretching vibrations. It also includes O-H stretching vibrations from bound and residual water. The broad shape reflects strong hydrogen bonding in the gelatine matrix. Gelatine is hygroscopic and retains moisture easily. However, both samples were dried and stored under identical conditions. Therefore, moisture effects are comparable and do not affect relative interpretation.

Both samples also showed features of the Amide B band. CBBG5 showed additional shoulders at 2,927 cm^-1^ and 2,848 cm^-1^. These peaks are related to asymmetric and symmetric C--H stretching vibrations. Their presence suggests that higher acetic acid concentration increases structural modification. It also affects the hydrophobic environment of the gelatine matrix (Wei et al., 2024).

FTIR confirms the presence of all major functional groups in both samples. These include Amide A, Amide I, Amide II, Amide III, and Amide B. This confirms successful gelatine extraction. However, differences in peak positions and intensities were observed. These differences indicate that higher acetic acid concentration causes greater disruption of protein structure. This affects secondary structure and hydrogen bonding. These findings agree with previous studies on extraction-condition effects on gelatine structure (Lv et al., 2019; Yu et al., 2023).

### 3.3 Morphological analysis from electron microscopy

SEM analysis was performed to assess the effect of acetic acid concentration on the structural properties of gelatine extracted from chicken by-products. Two types of samples were examined: gelatine powder and gelatine gel, with CBBG3 prepared using 3 v/v% acetic acid and CBBG5 using 5 v/v% acetic acid. The SEM images of both forms revealed differences in particle size and gel network structure related to the acetic acid concentration used during extraction. However, residual bound water in gelatine may influence SEM surface morphology through vacuum-induced micro-evaporation, leading to localized structural relaxation and surface smoothing effects during imaging.

**FIGURE 3:**
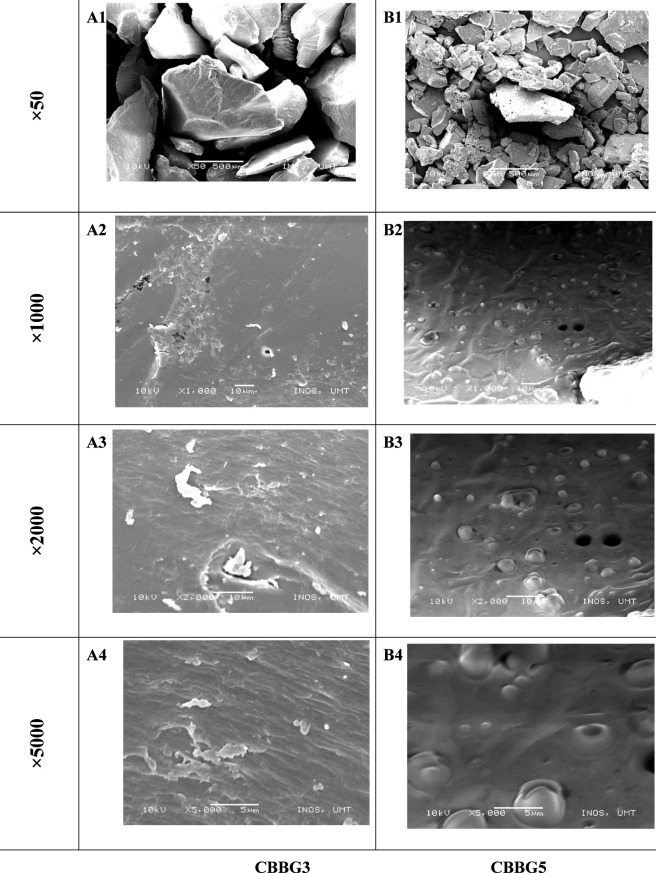
SEM images of gelatine powders extracted from chicken heads and feet **(A1-A4)** CBBG3: Gelatine extracted with 3v/v% acetic acid, showing larger particles with a smoother, more uniform surface **(B1-B4)** CBBG5: Gelatine extracted with 5v/v% acetic acid, exhibiting smaller particles with a more irregular, bubbly surface. The powders were prepared from samples pretreated with 0.5 M NaOH, soaked in acetic acid, heated at 75 °C, dried, and ground into powder.

Figure 3 presents the SEM images of the gelatine powders. CBBG3, which was extracted with 3v/v% acetic acid, exhibits larger particles with a smoother, more uniform surface (Figures 3A1–A4). This smooth surface indicates that the lower acetic acid concentration causes less disruption to the collagen structure, preserving its integrity and resulting in a more uniform particle size. In contrast, CBBG5, extracted with a higher concentration of 5v/v% acetic acid, shows smaller particles with a more irregular, bubbly surface (Figures 3B1–B4). The higher acetic acid concentration leads to increased cleavage of peptide bonds and more extensive breakdown of the collagen structure during pretreatment. This disruption results in smaller particles and a more porous surface. The observed differences in particle size and surface morphology align with findings from previous studies, which indicate that higher acid concentrations can lead to more significant degradation of collagen and a subsequent decrease in particle uniformity (Lin et al., 2024; Mudhafar et al., 2024).

**FIGURE 4:**
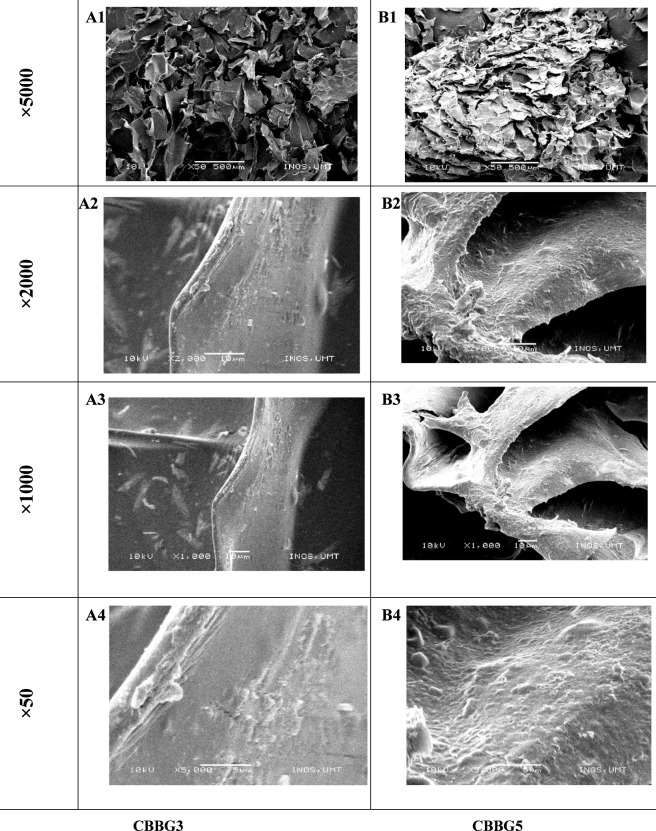
SEM images of gelatine gels prepared from powders dissolved in distilled water at a 5v/v% acetic acid, poured into Molds, set at 4 °C, and then analysed **(A1–A4)** CBBG3: Gelatine gel extracted with 3v/v% acetic acid, showing a finer, more densely cross-linked network with smaller pores **(B1–B4)** CBBG5: Gelatine gel extracted with 5v/v% acetic acid, displaying a coarser structure with larger voids and a less ordered network.

Figure 4 shows the microstructure of the gelatine gels prepared from the same by-products. The CBBG5 gelatine gel has a coarser structure with larger voids (Figures 4B1–B4), reflecting a less ordered network. This result is consistent with the higher acetic acid concentration used during pretreatment, which promotes greater breakdown of the collagen and affects gel formation by creating a less cohesive network. Conversely, CBBG3 displays a finer, more densely cross-linked network with smaller pores (Figures 4A1–A4). This more homogeneous gel structure is due to the milder pretreatment conditions, which better preserve the collagen’s structural integrity and result in a more ordered and durable gel network. The observed denser network and smaller pore size in CBBG3 contribute to its higher gel strength and improved textural properties, aligning with previous research. According to Wu et al. (2023) and Nandiyanto et al. (2019), gelatines with a finer and denser gel network have higher strength. This finding is consistent with the higher gel strength of CBBG3 gelatine and its better textural profile, as previously reported by Aidat et al. (2023). Consistent with other studies, the presence of a homogeneous structure of microscopic pores in the gelatine gel matrix is linked to the gelatine’s greater water absorption capacity (Liu et al., 2019; Mikhailov, 2023). Besides, the homogeneous structure of CBBG3 gelatine could indicate enhanced durability of its gel.

### 3.4 Protein profile using SDS-PAGE

SDS-PAGE analysis is essential for elucidating the protein profile of gelatine, providing insights into the molecular weights and relative abundances of various protein chains. This technique separates proteins based on size, allowing for detailed characterization of gelatine’s key components.

**FIGURE 5:**
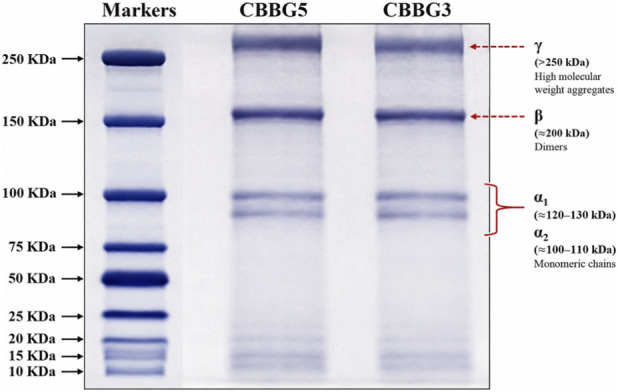
SDS-PAGE profiles of gelatines extracted from a mixture of chicken by-products (feet and heads). CBBG3: Gelatine extracted using 3v/v% acetic acid, showing characteristic bands for α-chains and β-chain. CBBG5: Gelatine extracted using 5v/v% acetic acid, displaying different band intensities and molecular weights.

Figure 5 displays the SDS-PAGE patterns of gelatines (CBBG3 and CBBG5), showing distinct protein profiles. Both samples exhibit the typical features of type I gelatine, characterized by three main bands: two α-chains (α1 and α2) and a β-chain representing their cross-linked dimer. CBBG3 gelatine shows higher molecular weights for the α-chains, approximately 156 kDa for α1 and 130 kDa for α2. In contrast, CBBG5 gelatine presents α-chains with lower molecular weights of about 137.1 kDa and 82.6 kDa, respectively. This observation supports the findings of Gomez-Guillen et al. (Wang et al., 2023), who reported that higher α-chain content is associated with better functional properties, aligning with the higher quality of CBBG3 gelatine as suggested by previous studies (Aidat et al., 2023).

Moreover, CBBG3 gelatine prominently features a 250 kDa γ-chain band, which is absent in CBBG5. This indicates that the 3v/v% acetic acid pretreatment is more effective in extracting gelatine with enhanced molecular stability. The presence of the γ-chain suggests improved structural integrity and potential functional benefits, such as superior gelling and binding properties. This finding contrasts with studies on gelatines extracted from chicken feet (Erg et al., 2018; Santana et al., 2020), and heads (Ee et al., 2021; Gál et al., 2020), where the γ-chain was not detected, highlighting the unique advantages of the 3v/v% acetic acid pretreatment in producing higher-quality gelatine.

### 3.5 Thermal behaviour from DSC analysis

**FIGURE 6:**
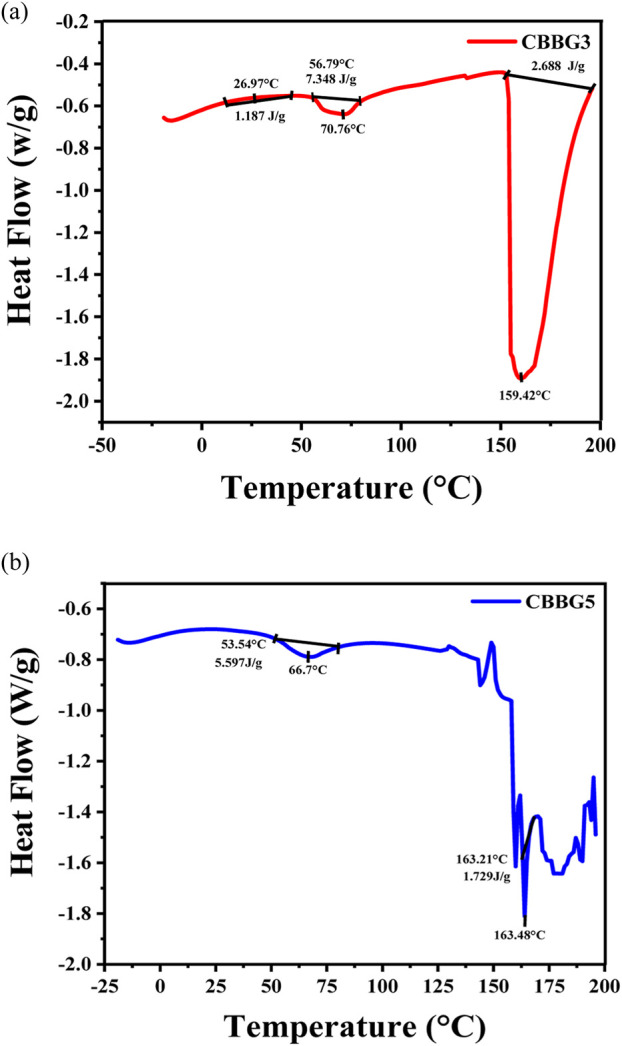
DSC curves for CBBG3 and CBBG5 gelatines, highlighting their distinct thermal behaviours. **(a)** CBBG3: Gelatine extracted using 3v/v% acetic acid. **(b)** CBBG5: Gelatine extracted using 5 v/v% acetic acid.

Thermal behaviour analysis is essential for assessing gelatine stability when exposed to heat, particularly its denaturation behaviour during heating. DSC provides critical insights into the thermal properties of gelatine, including the glass transition temperature (Tg), transition enthalpy (ΔH), and heat capacity (Du et al., 2021). Figure 6 presents the DSC thermograms of CBBG3 and CBBG5 gelatines, highlighting their distinct thermal behaviours. The Tg of CBBG3 was observed at 26.97 °C, whereas CBBG5 did not exhibit a distinct Tg peak, indicating that CBBG3 possesses greater resistance to temperature fluctuations. This observation is consistent with the findings of Castro et al. (2022), who reported that higher Tg values are associated with enhanced thermal stability in gelatine systems.

Following Tg, CBBG3 exhibited two endothermic peaks at 70.76 °C and 159.42 °C, while CBBG5 showed multiple endothermic peaks at 66.77 °C, 163.21 °C, and 163.48 °C. The endothermic transition occurring immediately after Tg in both samples corresponds to the helix-to-coil (gel-sol) transition of the gelatine network, associated with the thermal dissociation of triple-helical structures (Leyva-Porras et al., 2019; Wang et al., 2022). At higher temperatures, degradation was observed as a single peak at 159.42 °C for CBBG3 and as multiple peaks around 163.21 °C–163.48 °C for CBBG5, reflecting peptide bond cleavage and the onset of gelatine thermal degradation (Castro et al., 2022).

These results indicate that CBBG3 gelatine exhibits superior thermal behaviour compared to CBBG5, likely due to more effective extraction and pretreatment conditions. The transition enthalpy (ΔH) further differentiates the two samples, with CBBG3 displaying a higher value (2.688 J g^-1^) than CBBG5 (1.729 J g^-1^). The higher ΔH value suggests a greater degree of molecular order and enhanced thermal stability of the gelatine network, which may be attributed to its lower ash content (Saenmua et al., 2020) and higher Bloom strength (Aidat et al., 2023). Increased Bloom strength is known to be associated with stronger intermolecular interactions and higher melting temperatures, thereby contributing to improved gelatine thermal stability.

Our results agree with previous studies have shown that acid concentration plays a crucial role in determining the gelatine’s molecular weight, gel strength, and thermal stability. For instance, Sántiz-Gómez et al. (2019) demonstrated that gelatine extracted from fish skin using 3v/v% acetic acid exhibited superior gel strength and higher molecular weight compared to gelatine extracted with 5 v/v% acetic acid, which resulted in a weaker gel with reduced thermal stability. Similarly, Amiza et al. (2015) found that lower concentrations of acetic acid (2 v/v% to 3 v/v%) in the extraction process led to gelatine with enhanced structural integrity and better functional properties, including higher viscosity and greater water-binding capacity, compared to higher concentrations which caused more protein degradation. These findings support our observation that 3v/v% acetic acid yields a higher quality gelatine with better structural stability and functional characteristics than 5 v/v% acetic acid. Similarly, the use of sulfuric acid in gelatine extraction has been associated with excessive breakdown of the gelatine structure, leading to poor-quality products with lower gel strength and altered functional properties (Mikhailov, 2023). These observations show the advantage of using milder acids like acetic acid for gelatine extraction, as highlighted in our study, which shows that controlled acetic acid concentrations effectively balance the need for efficient collagen extraction with the preservation of gelatine’s desirable properties.

## 4 Conclusion

This study provides a detailed comparison of gelatine extracted from chicken by-products using different concentrations of acetic acid, highlighting the significant impact of pretreatment conditions on gelatine properties. FTIR analysis confirmed that gelatine extracted with 3 v/v% acetic acid (CBBG3) has a more stable molecular structure, as evidenced by stronger amide bands. SEM imaging illustrated that CBBG3 has a smoother and more uniform particle surface compared to the rougher, more irregular surface of gelatine extracted with 5 v/v% acetic acid (CBBG5). The SDS-PAGE results showed that CBBG3 had higher molecular weights for α1 and α2 chains (156 kDa and 130 kDa) compared to CBBG5 (137.1 kDa and 82.6 kDa), suggesting superior functional properties. DSC analysis further demonstrated that CBBG3 has a higher glass transition temperature (26.97 °C) and a greater enthalpy of fusion (ΔH_f_, 2.688 J/g), indicating enhanced thermal stability compared to CBBG5, which showed no clear Tg and lower values for ΔH_f_ (1.729 J/g). These findings align with previous literature, suggesting that lower acetic acid concentrations yield gelatine with improved structural integrity, functional capabilities, and thermal resistance. Consequently, gelatine extracted with 3v/v% acetic acid is preferable for applications requiring higher stability and better performance, making it a more suitable option for various industrial and food applications compared to gelatine extracted with higher acetic acid concentrations.

## Data Availability

The original contributions presented in the study are included in the article/supplementary material, further inquiries can be directed to the corresponding author.
